# Comparative analysis of quantitative efficiency evaluation methods for transportation networks

**DOI:** 10.1371/journal.pone.0175526

**Published:** 2017-04-11

**Authors:** Yuxin He, Jin Qin, Jian Hong

**Affiliations:** School of Traffic and Transportation Engineering, Central South University, Changsha, Hunan, P.R. China; Beihang University, CHINA

## Abstract

An effective evaluation of transportation network efficiency could offer guidance for the optimal control of urban traffic. Based on the introduction and related mathematical analysis of three quantitative evaluation methods for transportation network efficiency, this paper compares the information measured by them, including network structure, traffic demand, travel choice behavior and other factors which affect network efficiency. Accordingly, the applicability of various evaluation methods is discussed. Through analyzing different transportation network examples it is obtained that Q-H method could reflect the influence of network structure, traffic demand and user route choice behavior on transportation network efficiency well. In addition, the transportation network efficiency measured by this method and Braess’s Paradox can be explained with each other, which indicates a better evaluation of the real operation condition of transportation network. Through the analysis of the network efficiency calculated by Q-H method, it can also be drawn that a specific appropriate demand is existed to a given transportation network. Meanwhile, under the fixed demand, both the critical network structure that guarantees the stability and the basic operation of the network and a specific network structure contributing to the largest value of the transportation network efficiency can be identified.

## Introduction

Transportation network efficiency as a comprehensive reflection of the operation of transportation networks, is primarily determined by its network structure, by the volume and distribution of travel demand, and by drivers’ routing behavior. Transportation network efficiency has become one of the focal problems in transportation network research and practical work in recent years. It is generally defined as the degree of users satisfaction with the certain amount of transportation investments to traffic demand. But this definition cannot completely reflect the effects of network topology, travel choice behaviors, traffic demands and travel costs on transportation network efficiency. In fact, the connotation of the transportation network efficiency is multi-level, the consideration and evaluation of the transportation network efficiency are different according to different stakeholders, different system objectives and different research perspectives. Depending on the characteristics of research methods, the current study of the transportation network efficiency evaluation can be divided into qualitative research and quantitative research.

In qualitative research, transportation network efficiency evaluations are mainly based on the multi-index evaluation method. Costa and Markellos analyzed the production efficiency of urban public transport services, but in fact they analyzed the competitive efficiency of the bus enterprises instead of the network operating efficiency[1]. Levinson got an all-round understanding of transportation network efficiency from flexibility, efficiency, accessibility, productivity, utilization fairness five aspects[2]. Nait-Sidi-Moh, Manier and Moudni evaluated the performance of bus network and considered the passengers’ waiting time as the unique indicator to measure the performance of the public transportation network. In fact, they evaluated the coordination of vehicles on the different lines in the intersection of the public transportation network, rather than the efficiency of the entire network[3]. The multi-index qualitative evaluation methods above could reflect the operational efficiency of the transportation network to a certain extent, but there are too many subjective factors during the evaluation process to ensure the objectivity and rationality of the evaluation results. In addition, some scholars used data envelopment analysis or stochastic frontier analysis methods to study the input-output efficiency of the transportation network, which was actually the relative efficiency of input-output ratio of the enterprise rather than the network operation efficiency, the studies of Sveinn, Ming-Miin and Erwin were such kind of research[4,5].

In quantitative research, the efficiency of transportation networks refers to the quantity of traffic demands that can be met through a certain amount of investments in travel costs (network impedance) within the transportation network. Scholars with the represent of Huang carried out a series of in-depth researches at this basis and achieved fruitful research results, but only using the network impedance as the unique indicator to measure the network efficiency is obviously not comprehensive[6]. Latora and Marchiori presented a quantitative method to calculate the weighted network efficiency, and used the shortest distance between any two points in the network as a parameter for network efficiency evaluation and applied it in complex network in their Related studies[7–13], but this approach failed to consider the effects of road congestion, therefore it could not be directly used to measure the efficiency of the transportation network; Hsu and Shih also used this method to analyze the aviation network efficiency, although it was not necessary to consider the aviation network crowding effect, but the authors did not consider other factors affecting the network efficiency such as the traffic demand [14]. Nagurney and Qiang proposed a quantitative measure for congested network based on the path impedance and demand, which did not consider the impact of the transportation network size on the efficiency, but also did not perform in-depth analysis of the efficiency’s characteristics[15–17]. Qin and He proposed a new quantitative evaluation method for the transportation network efficiency, calculating the transportation network efficiency by using objective indicators which are link traffic flows and link impedance as parameters. This method can reflect the effects of the network structure, traffic demand and user route choice behavior on transportation network efficiency. They also found that the transportation network efficiency calculated by this method could explain Braess’s Paradox in transportation networks, which also proved the reasonability of this method [18].

In general, the research on the transportation network efficiency are mainly based on subjective and qualitative analysis, while objective and quantitative evaluation mostly could not be used directly in transportation networks with the crowding effect, especially the research of quantitative evaluation methods for transportation network efficiency which could be used in a mathematical model are only at the primary stage. In the following sections of this paper, three typical quantitative efficiency evaluation methods for transportation networks are introduced and analyzed comparatively.

## Quantitative efficiency evaluation methods for transportation networks

A transportation network is considered as *G* = (*N*,*A*), in which *N* is the set of nodes and consists of *n* elements, *A* is the set of links with *n*_*A*_ elements. *W* is the set of OD pairs of nodes with *n*_*W*_ elements. The set of paths connecting OD pair *w* ∈ *W* is denoted by *K*_*w*_. The demand for OD pair w is denoted by *q*_*w*_. The flow and travel cost on link *a* ∈ *A* are *x*_*a*_ and *t*_*a*_(*x*_*a*_), respectively. The flow on path *k* ∈ *K*_*w*_ is denoted by $f_{w}^{k}$. Decision variables defined as follows:

$$\delta_{w}^{a,k}=\left\{ \begin{array}{l} 1\;if\;l\;ink\;a\;is\;on\;path\;k\;connecting\;OD\;pair\;w;\\ 0\;otherwise\; \end{array}\right.$$

In recent years, there have been plentiful research on traffic equilibrium problems under different conditions, but the existing quantitative evaluation methods for transportation networks still mainly focus on the traffic flows at equilibrium and other factors, therefore this paper carries out related research and analysis based on the UE (User Equilibrium) model under fixed demand.

UE model with a fixed demand is described as follows:

$$\min\;Z(x)=\sum_{a\in A}\int_{0}^{x_{a}}t_{a}(y)d_{y}$$ (1)

$$s.t.\;\sum_{k\in K_{w}}f_{w}^{k}=q_{w}\;,\forall w\in W$$ (2)

$$x_{a}=\sum_{w\in W}\sum_{k\in K_{w}}f_{w}^{k}\delta_{w}^{a,k},\;\forall a\in A$$ (3)

$$f_{w}^{k}\geq 0,\;\forall w\in W,k\in K_{w}$$ (4)

In this model, the objective function Eq (1) is to minimize the total travel costs in the network. The function does not have any economic or behavioral interpretation, and it should be viewed as a mathematical concept that is utilized to solve equilibrium problems. Eq (2) represents a set of flow conservations, which means that the sum of flows on paths connecting each OD pair w must be equal to the demand between OD pair *w*. In other words, all OD demands have to be assigned to the network. The link flows are related to the path flows through the conservation of flow Eq (3), that is, the user cost on a path is equal to the sum of user costs on links that make up the path. Eq (4) is the nonnegative constraint on path flows, and it ensures that the solution of the model will be physically meaningful.

The solutions of the above model are relatively mature, this paper selected computationally efficient gradient projection (Gradient Projection, GP) algorithm to solve the above model, The details of the GP method can be found in Qin et al.(2013) and Chen et al.(2002)[19, 20].

As mentioned earlier, the current quantitative efficiency evaluation methods can be applied to urban transportation networks, mainly proposed by Latora and Marchiori(2001), Nagurney and Qiang (2007), and Qin and He (2014). The later descriptions are denoted by L-M, N-Q and Q-H methods, these three methods are described as follows:

a L-M method

Latora and Marchiori proposed “global efficiency” to evaluate network performance in a weighted network. The method could be recalled as follows [7]:

$$E_{LM}=\frac{1}{n_{N}(n_{N}-1)}\sum_{i\neq j\in G}\frac{1}{d_{ij}}\;$$ (5)

Where *d*_*ij*_ is the shortest path length between the node *i* and node *j*, it can be understood as the travel time at equilibrium between the two nodes.

b N-Q method

Nagurney and Qiang put forward a network efficiency measure for congested networks, which can be calculated as follows[15]:

$$E_{NQ}=\frac{1}{n_{W}}\sum_{w\in W}\frac{q_{w}}{t_{w}}\;$$ (6)

Where *t*_*w*_ is the shortest travel time between OD pairs *w* ∈ *W*, it can be understood as transportation network path travel time at equilibrium between OD pairs *w*.

c Q-H method

Qin and He proposed a new quantitative evaluation method for transportation network efficiency reflecting the effects of the network structure, traffic demand and user route choice behavior, which is calculated as follows[18]:

$$E_{QH}=\frac{1}{n_{A}}\sum_{a\in A}\frac{x_{a}}{t_{a}}\;$$ (7)

Where *x*_*a*_ and *t*_*a*_ are the flow and travel costs on link *a* at equilibrium respectively.

According to the above formulas (5)–(7), while calculating the efficiency with L-M method (5), it only takes into account shortest travel time between the two nodes and the number of nodes. Since the link travel time of the network is bound to increase as traffic increases, *E*_*LM*_ will reach the maximum when the flows on all links are 0. Obviously, *E*_*LM*_ is a monotone decreasing function of the demand, which is not in accord with actual conditions. In fact, *E*_*LM*_ represents the average distance between any two nodes in networks, that is, characteristic path length, which is defined as the average value of the path length of all node pairs in networks. and it is the global feature of networks. L-M method focused on giving a clear physical meaning of the small world behavior, which can conduct the quantitative analysis of the information flow accurately, but the features of transportation networks cannot be reflected accurately through L-M method. Hence it is concluded that L-M method is more suitable for the analysis of the small world behavior of networks and characteristics of communication networks.

When calculating network efficiency with N-Q method (6), factors considered are the number of OD pairs, traffic demand and the path travel time, but this method fails to reflect the direct impact of the network structure or the topology on the transportation network efficiency.

The network efficiency calculated with Q-H method (7) takes into account the impact of network structure, link travel time and traffic demand. The rationality and applicability of the transportation network efficiency calculation methods above will be further discussed in the following numerical examples analysis.

## Comparison and analysis of network efficiency evaluation methods

In this section, we use three numerical examples to calculate and compare the three methods above.

### Validation example

**Fig 1:**
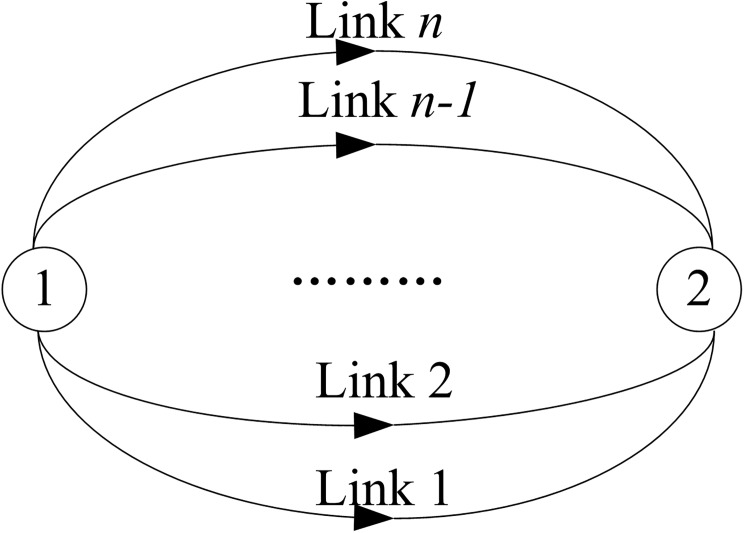
Validation network.

Fig 1 shows a simple transportation network with only two nodes. The network has only one OD pair (1,2), the traffic demand is *q*. Assume that there are *n* links connecting between nodes 1 and 2, and the travel time of all the paths is the same, calculated by BPR function, that is, $t(x)=t_{0}(1+{\alpha (\frac{x}{c})}^{\beta })$, where *t*(*x*) is the link travel time, *x* is the traffic flow on the link, *t*_0_ is the free flow time, *c* is the traffic capacity, *α* and *β* are the given parameters.

According to the previous efficiency calculation Eqs (5)–(7), the three network efficiency can be calculated as follows:

$$E_{LM}=\frac{1}{2t(x)}$$ (8)

$$E_{NQ}=\frac{q}{t(x)}$$ (9)

$$E_{QH}=\frac{x}{t(x)}$$ (10)

Obviously, since all the travel time functions of the links are the same, the traffic flow on each link at equilibrium is necessarily equal, which is x=qn.

Accordingly, we take the derivative of formulas (8)–(10) with respect to the number of links *n* respectively, the results can be obtained as follows:

∂ELM∂n=αβnβ−12t0(nβ+αqβcβ)2 (11)

∂ENQ∂n=qt0αβnβ−1(nβ+αqβcβ)2 (12)

∂EQH∂n=qt0nβ−2(α(β−1)qβcβ−nβ)(nβ+αqβcβ)2 (13)

Since the parameters *α*,*β*,*c*,*t*_0_,*n*,*q* > 0, it can be easily drawn that $\frac{\partial E_{LM}}{\partial n}>0$, $\;\frac{\partial E_{NQ}}{\partial n}>0$. It means that when *q* is constant, the efficiency *E*_*LM*_ and *E*_*NQ*_ are increasing functions of the links number *n*, that is, the more links of the network, the higher network efficiency will be. Obviously, it does not conform to the objective law of transportation networks operation.

With regard to $\frac{\partial E_{QH}}{\partial n}$, assuming the parameter *β* > 1 (Generally *β* = 4 in actual calculation), through further calculation, it can be found that, when $n<\sqrt[{\beta }]{\alpha (\beta -1)q^{\beta }/c^{\beta }}$, $\frac{\partial E_{QH}}{\partial n}>0$, *E*_*QH*_ will increase with the number of links *n* increasing, when $n>\sqrt[{\beta }]{\alpha (\beta -1)q^{\beta }/c^{\beta }}$, $\frac{\partial E_{QH}}{\partial n}<0$, *E*_*QH*_ will decrease with the number of links *n* increasing, when $n=\sqrt[{\beta }]{\alpha (\beta -1)q^{\beta }/c^{\beta }}$, *E*_*QH*_ will reach the maximum. It can be explained that when the network demand is constant, the network efficiency will increase then decrease as the number of links increasing. This change law also shows *E*_*QH*_ is associated with the number of links *n*, link capacity *c*, traffic demand *q*, and even parameters *α*,*β*.

**Fig 2:**
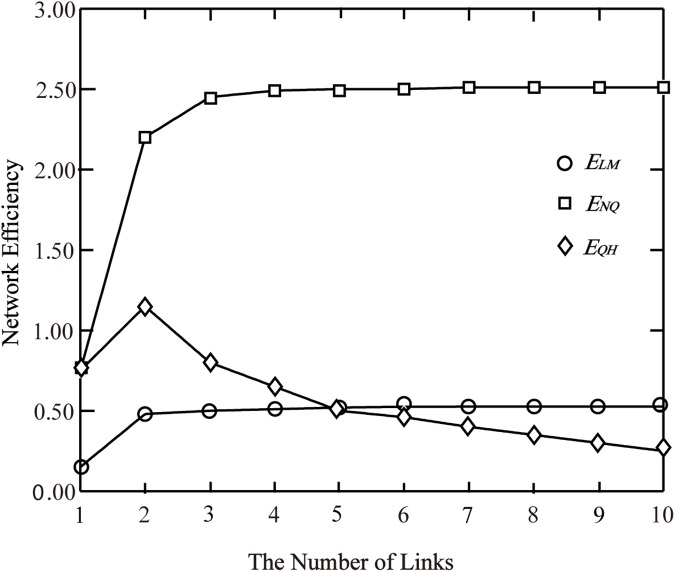
The network efficiency vs. the number of links.

Then, given network parameters are as follows: *q* = 10, travel time function of all links are $t(x)=4(1+0.15{\left(\frac{x}{5}\right)}^{4})$, therefore the rules of the efficiency changing with the number of links *n* can be shown in Fig 2.

With regard to the network efficiency *E*_*QH*_, according to the previous analysis, *E*_*QH*_ reaches the maximum when n=0.15×(4−1)×104544=1.638, considering that *n* is the number of links, which is rounded as *n* = 2. The change of *E*_*QH*_ in Fig 2 accords with the analysis results.

Through the comparative analysis in the validation network, it is known that when demand remains constant, *E*_*LM*_ and *E*_*NQ*_ present the monotonically increasing trend with the gradual expansion of the transportation network scale, while *E*_*QH*_ presents a trend of rise first then fall, which is resulted from the number of links, link capacity, demand and other factors.

The larger the network scale and capacity are, the shorter user travel time is. It is clearly beneficial from the user’s point of view. But from the view of the whole transportation network systems, for larger network scale and capacity, fewer users served will whereas make the overall operation of the network more efficient, which is obviously unreasonable. The fluctuation of *E*_*QH*_ affected by many factors compared with monotonic increasing of *E*_*LM*_ and *E*_*NQ*_, and it is in line with the actual conditions of the transportation network efficiency. From the analysis of validation network, it can be found that *E*_*QH*_ is more reasonable to describe transportation networks operating efficiency. This conclusion needs to be further discussed and verified in the following more complex networks.

### Transportation network example 1

**Fig 3:**
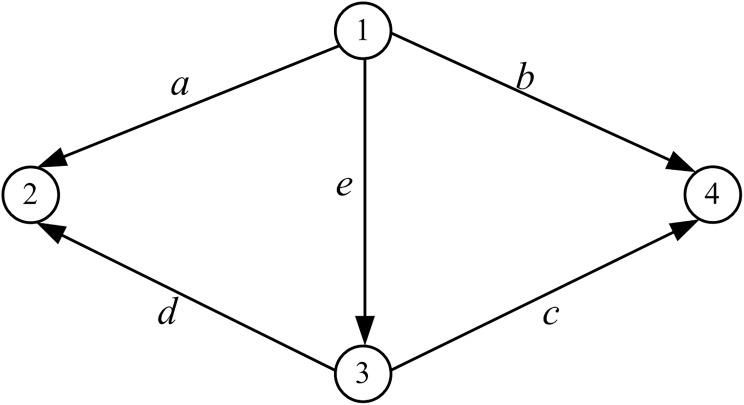
Transportation network 1.

In a simple transportation network as shown in Fig 3, there are four nodes, five links and five OD pairs: (1,2), (1,4), (1,3), (3,2) and (3,4), the demands are *q*_12_ = 11, *q*_14_ = 6, *q*_13_ = 2, *q*_32_ = 3, *q*_34_ = 1, respectively. S1 Table. The link cost functions are set as follows, and in S2 Table.

ta(xa)=10(1+0.15(xa4)4),tb(xb)=15(1+0.15(xb6)4),tc(xc)12(1+0.15(xc3)4),td(xd)=15(1+0.15(xa10)4),te(xe)=20(1+0.15(xe8)4).

The traffic flow of network links at equilibrium can be obtained easily by using GP algorithm, which is:

$$x=\{x_{a},x_{b},x_{c},x_{d},x_{e}\}=\{7.813,5.762,1.238,6.187,5.425\}$$

In this case, the network efficiency calculated by Eqs (5)–(7) are as follows: *E*_*LM*_ = 0.0236, *E*_*NQ*_ = 0.2068, *E*_*QH*_ = 0.2563. At this point, the total travel cost of the network is 120.1734.

**Fig 4:**
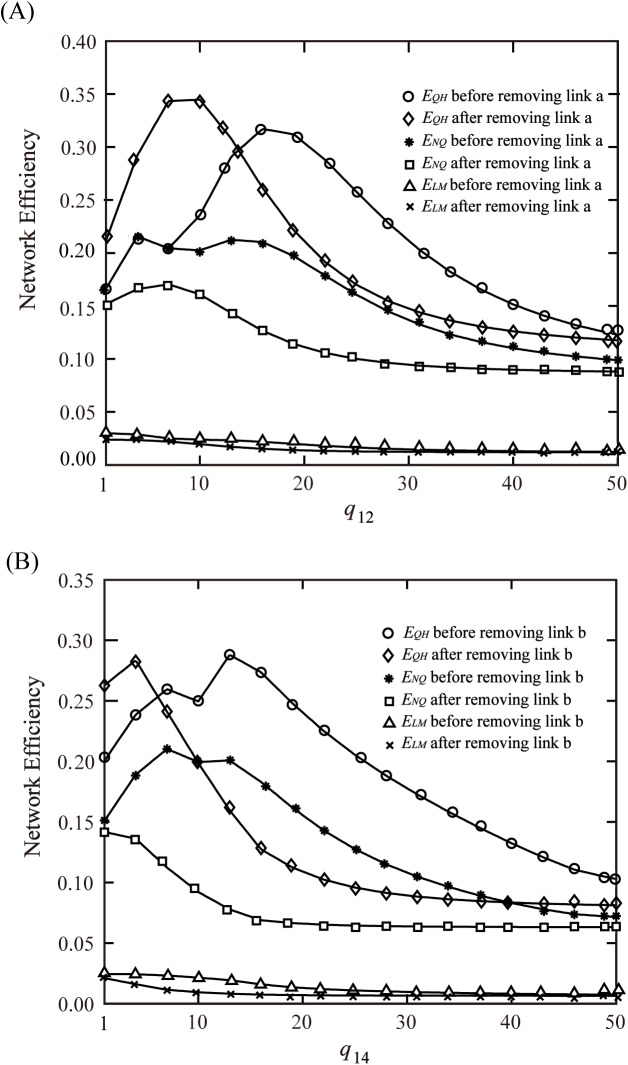
Efficiency changes with demands changing. (A) Efficiency changes with *q*_12_ before and after removing link a. (B) Efficiency changes with *q*_14_ before and after removing link b.

Through analyzing the network efficiency before and after the link break or damage of the network, the key link in the network can be found. For example, Du analyzed the network robustness before and after removing certain links, and found the set of vital links[21, 22]. It proves that this analysis method is effective. The following Fig 4 shows that the network efficiency changes with the demands changing before and after removing links.

It is confirmed that the efficiency *E*_*LM*_ still remains monotonically decreasing with the increasing of the demand in a complex transportation network. *E*_*LM*_ before removing the link is always bigger than the one after removing the link, and it accords with the conclusions of the previous analysis, that is, *E*_*LM*_ is an increasing function of the network size. Accordingly, it is noted that *E*_*LM*_ cannot describe how demand and network structure affect network performance.

In contrast with the changing pattern of *E*_*LM*_, *E*_*NQ*_ and *E*_*QH*_ present the fluctuating variation with the changing of network structure and demands. With regard to *E*_*NQ*_ and *E*_*QH*_ before removing the link *a*, the changing laws have some similarities, they both have two extreme points. It results from there being two connected paths between the OD pairs (1,2) and (1,4), for distributing flows of corresponding demands. However, after removing link a or b, there is only one connected path between the OD pairs (1,2) or (1,4), herein *E*_*NQ*_ presents monotonically decreasing trend as the demand increasing, it reaches maximum when the demand is 0, and *E*_*QH*_ shows the trend of firstly rising then declining, and there is still an extreme point.

In Fig 4A and 4B, it can also be found that *E*_*NQ*_ after removing links is always smaller than that before removing links, which also proves that *E*_*NQ*_ is the monotonically increasing function of network size. On the contrary, as for *E*_*QH*_, the value after removing link a or link b is larger than the value before removing links within a specific range of demands, which is known as Braess’s Paradox in transportation networks, that is, the improving of the link capacity will lead to network efficiency decreasing instead[23].

According to the analysis above, it could be concluded that *E*_*LM*_ is a monotonically decreasing function of the network demand, and *E*_*NQ*_ and *E*_*QH*_ present the fluctuating changes with the network demands changing. Additionally, there is a demand appropriate to a given transportation network. *E*_*QH*_ can be used to explain Braess’s Paradox in transportation networks, thus it appears more reasonable by principle.

### Transportation network example 2

**Fig 5:**
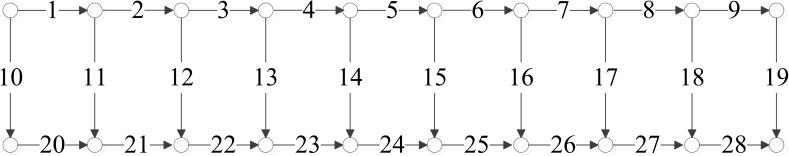
Transportation network 2.

Considering a transportation network in Fig 5, there are 20 nodes and 28 links in total. The network has only one OD pair: (1,20). The demand is given by: *q*_1,20_ = 100. Assuming the link travel cost function also adopts BPR function, that is, $t(x)=t_{0}(1+\alpha {\left(\frac{x}{c}\right)}^{\beta })$, wherein, the free flow time *t*_0_ of each link and the link capacity c are given in the following Table 1, and in S3 and S4 Tables.

**Table 1:** The basic attributes of network.

Link	*t*_0_	*C*	Link	*t*_0_	*C*
1	20	5	15	8	9
2	8	4	16	12	8
3	14	3	17	18	7
4	16	6	18	12	5
5	24	6	19	24	8
6	20	7	20	12	6
7	16	8	21	16	4
8	26	5	22	20	6
9	28	6	23	14	9
10	32	4	24	16	8
11	26	7	25	18	9
12	28	8	26	12	7
13	24	7	27	20	8
14	20	8	28	26	7

Here, when the demand *q*_1,20_ is constant, considering one path between the OD pair (1,20):1-2-3-4-5-6-7-8-9-10-20, we explore the changes of *E*_*LM*_, *E*_*NQ*_, *E*_*QH*_ with removing the links of the path one by one. In order to simplify description, let the notations in Table 2 denote different states of the link-removed network structure.

**Table 2:** Notations of network structure.

Notations	Network structure
X_1_	The initial network structure.
X_2_	The network that link 19 was removed.
X_3_	The network that link 9 and 19 were removed.
X_4_	The network that link 8,9,19 were removed.
X_5_	The network that link 7,8,9,19 were removed.
X_6_	The network that link 6,7,8,9,19 were removed.
X_7_	The network that link 5,6,7,8,9,19 were removed.
X_8_	The network that link 4,5,6,7,8,9,19 were removed.
X_9_	The network that link 3,4,5,6,7,8,9,19 were removed.
X_10_	The network that link 2,3,4,5,6,7,8,9,19 were removed.
X_11_	The network that link 1, 2,3,4,5,6,7,8,9,19 were removed.

**Fig 6:**
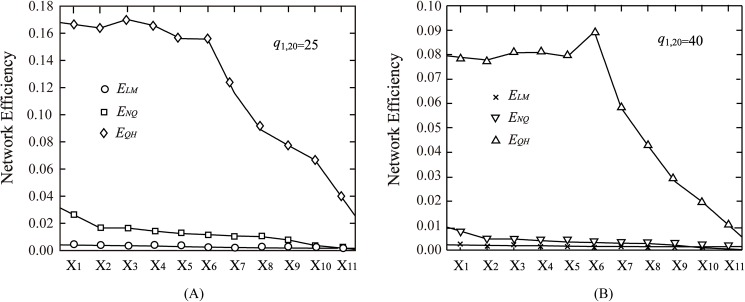
Network efficiency vs network structure. In the case of *q*_1,20_ = 25. (B) In the case of *q*_1,20_ = 40.

The calculation results are as follows in Fig 6. As the results showed in figures, in the transportation network example 2, *E*_*LM*_ firstly decreases then tends to be constant, and with the more links removed, the smaller *E*_*LM*_ is, which is consistent with the previous conclusion that *E*_*LM*_ is an increasing function of link numbers.

When *q*_1,20_ = 25, *E*_*NQ*_ falls to 0.0264 after removing link 19, then the efficiency value keeps 0.0165 after removing links 9 and 19, because both the link 9 and link 19 are in and only in one same path between the OD pair, without affecting the connection of other paths after removing them, therefore *E*_*NQ*_ is constant. Additionally, after removing more links, the value decline of *E*_*NQ*_ is barely measurable. Besides, for one path between the OD pair, the path is disconnected by just removing one link, at this time *E*_*NQ*_ decreases sharply, then the change of *E*_*NQ*_ is minor after removing more links of the path, which cannot reflect the change of the network structure, so it could be considered that *E*_*NQ*_ is not be able to reflect the information about the structure of the network.

According to the change of *E*_*QH*_, it shows that *E*_*QH*_ is not the monotonic function of the number of links. When *q*_1,20_ = 25, *E*_*QH*_ falls to 0.1638 after removing link 19, nevertheless, after removing links 9 and 19 it rises to the extreme point, 0.1702. This is because with the demand *q*_1,20_ = 25, a path between the OD pair (1, 20) is no longer connected after removing the link 19, the presence of the link 9 did not have an effect on the distribution of flow, so the efficiency value will increase after removing links 9 and 19.

In addition, the efficiency of X_3_ is the maximum under the current demand, which shows that this network structure is the most appropriate structure for the current demand. When *q*_1,20_ = 40, *E*_*QH*_ also falls to 0.0778 after removing link 19, then rises to 0.0807 after removing links 9 and 19, until after removing links 6,7,8,9,19, *E*_*QH*_ reaches the maximum 0.0893, which shows that X_6_ is the most appropriate network structure. Therefore, it can be considered that according to different demands, the most appropriate structures of transportation network are also different.

Additionally, as the network structure changes from X_6_ to X_7_, that is, as removing links 5,6,7,8,9,19 and even more links, *E*_*QH*_ begins to drop off sharply, it mainly because that the more links were removed, the fewer paths connected between OD pair (1, 20), and the number of connected paths is reduced from 6 to 5, therefore the network cannot accomplish the distribution of traffic flows, thus leads to the rapidly decreasing of the network efficiency value. Specifically, with the network structure changes from X_6_ to X_7_, when *q*_1,20_ = 25, *E*_*QH*_ greatly reduces from 0.156 to 0.116, when *q*_1,20_ = 40, *E*_*QH*_ also decreases rapidly from 0.089 to 0.058. And with the increase of deleted links, the efficiency continues to decrease substantially, until the network structure reaches X_11_, there is only one path connected between OD pair (1, 20), and efficiency reaches the minimum.

Therefore, according to network 2, X_6_ is the simplest network to satisfy the basic operation efficiency with *q*_1,20_ = 25 and *q*_1,20_ = 40, if removing more links on this foundation, it cannot satisfy the stability and normal operation efficiency of the network. Therefore, it can be found that, under the fixed demand, there is a network structure as the critical status to meet the basic operation efficiency and stability. On the basis of this case, some theoretical guidance can be added to the analysis of the network structure rationality.

In conclusion, as the results showed in the graph of *E*_*QH*_, the network efficiency does not totally depend on the number of links, it’s also influenced by the specific network structure. With a fixed demand, through calculating *E*_*QH*_ changing with the number of links and network structures, the specific network structure contributing to the largest value of the transportation network efficiency can be found. In addition, the critical network structure that guarantees the stability and the basic operation of the network can also be identified. Consequently, it can be noted that *E*_*QH*_ can reflect more information of network structure compared with *E*_*LM*_ and *E*_*NQ*_.

In summary, this paper combines with the related theoretical analysis and numerical calculation conclusions, the application scope of the three methods and their pros and cons are summarized as follows in Table 3:

**Table 3:** Comparison of three evaluation methods.

Method	L-M Method	N-Q Method	Q-S Method
**Network Efficiency**	$E_{LM}=\frac{1}{n_{N}(n_{N}-1)}\sum_{i\neq j\in G}\frac{1}{d_{ij}}\;$	$E_{NQ}=\frac{1}{n_{W}}\sum_{w\in W}\frac{q_{w}}{t_{w}}\;$	$E_{QS}=\frac{1}{n_{A}}\sum_{a\in A}\frac{x_{a}}{t_{a}}\;$
**Application Scope**	▪More suitable for the analysis of the information flow efficiency, ▪ Not applicable to congestion network efficiency evaluation.	▪Can be applied to the efficiency evaluation of congested network.	▪ Can be applied to the efficiency evaluation of congested network.
**Pros**	▪The accurate quantitative analysis can be given to the weighted networks and the non-weighted networks; ▪The evaluation of the information exchange efficiency of neural network and communication network is more accurate.	▪The comprehensive influence of traffic demand, travel cost and user's choice behavior on the network efficiency can be reflected.	▪Can reflect the comprehensive influence of the network structure, traffic demand, travel cost and the user's choice behavior on the network efficiency.
**Cons**	▪Not considering congestion effect, and cannot be directly applied to the evaluation of congestion network efficiency.	▪ Cannot reflect the influence of network structure on network efficiency.	▪ The computational complexity is relatively high.

## Conclusions

To conclude, this paper combines the related theoretical analysis with numerical calculation conclusions, the application scope of the three methods and their advantages and disadvantages are summarized. Among them, L-M method is more suitable for the analysis of the information network but not applicable to congestion network, since it does not consider congestion effect and cannot be directly applied to the evaluation of congestion network efficiency. N-Q method can be applied to the efficiency evaluation of congested network, and it could reflect the comprehensive influence of demand, travel cost and user's choice behavior on the network efficiency. However, it cannot reflect the influence of network structure on network efficiency.

Relative to these two network efficiency evaluation methods, Q-H method defined the transportation network efficiency from the perspective of the average cost of links, that is, the network efficiency is the average pay of the travel costs on unit link in the transportation network at equilibrium and the number of travelers can be served. The numerical examples proved that *E*_*QH*_ could reflect the effect of the network structure, demand and user route choice behavior on the transportation network efficiency. *E*_*QH*_ could explain Braess’s Paradox of transportation networks, and could truly reflect the actual operating situation of transportation networks. Therefore, it is noted that Q-H method can offer a reasonable evaluation index for the congested networks.

Besides, an intriguing feature of the transportation network efficiency can be concluded that the network efficiency will change with demands changing. For a given network structure, there is a most appropriate demand that the network can offer. In addition, the operation efficiency of transportation networks does not totally depend on the number of links, it also affected by the specific network structure, when demand is constant, there is a specific network structure contributing to the largest value of the transportation network efficiency. Meanwhile, the critical network structure that guarantees the stability and the basic operation of the network can also be identified under the fixed demand.

## Supporting information

S1 Table OD information of Transportation Network Example 1. (DOCX)

S2 Table Link attributes of Transportation Network Example 1. (DOCX)

S3 Table OD information of Transportation Network Example 2. (DOCX)

S4 Table Link attributes of Transportation Network Example 2. (DOCX)
