# Supplementary material for: Comparative analysis of quantitative efficiency evaluation methods for transportation networks
Source: PLoS One. 2017 Apr 11;12(4):e0175526. doi: 10.1371/journal.pone.0175526 (PMC5388484; doi:10.1371/journal.pone.0175526)
Supplement: S1 Table — (DOCX) [file pone.0175526.s001.docx]

**Comparative Analysis of Quantitative Efficiency Evaluation Methods for Transportation Networks**

Yuxin He, Jin Qin^*^ and Jian Hong

*School of Traffic and Transportation Engineering, Central South University, Changsha, Hunan, 410075, P.R. China*

| **OD No.** | **Origin** | **Destination** | **Traffic Demand *q*** |
| --- | --- | --- | --- |
| 1 | 1 | 2 | 11 |
| 2 | 1 | 4 | 6 |
| 3 | 1 | 3 | 2 |
| 4 | 3 | 2 | 3 |
| 5 | 3 | 4 | 1 |

**S1 Table**. OD information of Transportation Network Example 1.
