# Supplementary material for: Comparative analysis of quantitative efficiency evaluation methods for transportation networks
Source: PLoS One. 2017 Apr 11;12(4):e0175526. doi: 10.1371/journal.pone.0175526 (PMC5388484; doi:10.1371/journal.pone.0175526)
Supplement: S2 Table — (DOCX) [file pone.0175526.s002.docx]

**Comparative Analysis of Quantitative Efficiency Evaluation Methods for Transportation Networks**

Yuxin He, Jin Qin^*^ and Jian Hong

*School of Traffic and Transportation Engineering, Central South University, Changsha, Hunan, 410075, P.R. China*

| **Link** | $\boldsymbol{t}_{\mathbf{0}}$ | ***C*** |
| --- | --- | --- |
| a | 10 | 4 |
| b | 15 | 6 |
| c | 12 | 3 |
| d | 15 | 10 |
| e | 20 | 8 |

**S2 Table**. Link attributes of Transportation Network Example 1.
