# Supplementary material for: Comparative analysis of quantitative efficiency evaluation methods for transportation networks
Source: PLoS One. 2017 Apr 11;12(4):e0175526. doi: 10.1371/journal.pone.0175526 (PMC5388484; doi:10.1371/journal.pone.0175526)
Supplement: S4 Table — (DOCX) [file pone.0175526.s004.docx]

**Comparative Analysis of Quantitative Efficiency Evaluation Methods for Transportation Networks**

Yuxin He, Jin Qin^*^ and Jian Hong

*School of Traffic and Transportation Engineering, Central South University, Changsha, Hunan, 410075, P.R. China*

| **Link** | $\boldsymbol{t}_{\mathbf{0}}$ | ***C*** |
| --- | --- | --- |
| 1 | 20 | 5 |
| 2 | 8 | 4 |
| 3 | 14 | 3 |
| 4 | 16 | 6 |
| 5 | 24 | 6 |
| 6 | 20 | 7 |
| 7 | 16 | 8 |
| 8 | 26 | 5 |
| 9 | 28 | 6 |
| 10 | 32 | 4 |
| 11 | 26 | 7 |
| 12 | 28 | 8 |
| 13 | 24 | 7 |
| 14 | 20 | 8 |
| 15 | 8 | 9 |
| 16 | 12 | 8 |
| 17 | 18 | 7 |
| 18 | 12 | 5 |
| 19 | 24 | 8 |
| 20 | 12 | 6 |
| 21 | 16 | 4 |
| 22 | 20 | 6 |
| 23 | 14 | 9 |
| 24 | 16 | 8 |
| 25 | 18 | 9 |
| 26 | 12 | 7 |
| 27 | 20 | 8 |
| 28 | 26 | 7 |

**S4 Table**. Link attributes of Transportation Network Example 2.
